# Prism-based scanning X-ray microscopy

**DOI:** 10.1107/S2052252521008927

**Published:** 2021-09-01

**Authors:** Kenneth Evans-Lutterodt

**Affiliations:** a National Synchrotron Light Source II, Brookhaven National Laboratory, Building 743, PO Box 5000, Upton, NY 11973-5000, USA

**Keywords:** X-ray optics, X-ray nanoprobes, X-ray prisms, X-ray mirrors, scanning X-ray microscopy

## Abstract

A commentary is provided on a new type of prism deflection scanning X-ray microscope, providing context and some future potential applications of this new type of microscope.

In their letter in this issue of **IUCrJ**, Yamada *et al.* (2021 ▸) have pointed out a practical implementation of a new type of scanning X-ray microscope (SXM) with improved performance for some applications, based on scanning an X-ray spot across a sample, where the deflection of the beam is due to rotation of an X-ray prism, and the beam is focused to a spot by advanced Kirkpatrick–Baez (AKB) focusing mirrors. This in contrast with most existing synchrotron-based SXMs, in which the focused X-ray spot is fixed in space, and scanning is obtained by moving the sample (Kilcoyne *et al.*, 2003 ▸; Nazaretski *et al.*, 2015 ▸; Villar *et al.*, 2018 ▸).

Scanning spot microscopes play an important role and well established role in optical, electron and X-ray microscopies. One familiar and widely used laboratory instrument is the SEM (scanning electron microscope), in which one scans an electron beam across a sample, and the detector collects secondary electrons from the sample in order to construct the image. This is a close electron beam analog to the prism-based SXM implemented here. Scanned probes allow considerable flexibility in the type of signal detected, and the characteristics of detectors used. For the SXM implemented here, different types of detectors can be used in conjunction with the scanned X-ray beam, giving a wide range of modalities. A non-exhaustive list of observables includes wide-angle diffraction for crystal structure, fluorescence for elemental composition, ptychographic diffraction for imaging, X-ray beam-induced current and Compton scattering.

Existing (‘conventional’) SXMs today which scan the sample, use a complex optical/mechanical focusing assembly fixed in space to provide as small and as stable a focused X-ray spot as possible. Given the mechanical complexity of the incident beam optical path, moving the optics around to scan the beam is costly to implement, either fiscally or in experiment time, or both. For the case of a mirror-based optical path, the angles X-ray beams make with respect to the X-ray mirror surface are small, of order milli-radians, and consequently mirrors are long and bulky. Gravity sag is often sufficient to distort the mirror figure and this distorts the focused beam. It is in this context that the work of Yamada *et al.* (2021 ▸) is such an innovative advance where one can scan the focused spot, using a prism to deflect the beam prior to the optical focusing assembly, which can be kept fixed in space and optimally focused.

However, there are some issues that have to be addressed for this prism-based approach for the SXM to be effective. First, the deflection angles achievable by X-ray prisms are small. Roentgen’s initial measurements could not detect beam deflection, but later measurements by Compton (1923 ▸) and others (Compton, 1927 ▸) showed that the refractive index difference from vacuum was small, and thus practical deflection angles from a single prism are a fraction of the critical angle of the prism material, in this work approximately tens of micro-radians. In order for the prism-based scanning approach to be useful with such small deflection angles, one needs the focused beam sizes to be small. Recent advances in focusing optics as referenced in Yamada *et al.* (Yamauchi *et al.*, 2011 ▸; Mohacsi *et al.*, 2017 ▸; Bajt *et al.*, 2018 ▸) have provided significantly smaller X-ray beams, and have made the prism deflection approach practical and worth pursuing at this time.

A second consideration is that the focused spot profile should not change significantly as the deflection angle is changed. Here the team cleverly chose to use the more complicated AKB, instead of a regular KB system. The AKB consists of a combination of mirrors with elliptical and a hyperbolic profiles (Born & Wolf, 2001 ▸) and the reduced aberrations of this configuration, in comparison to a simple KB mirror, allow one to deflect the beam to larger angles with the AKB than is possible with the KB, with minimal spot size degradation.

An important advantage of the prism-based approach is that the prisms can be small and rigid, and this is amenable to high-speed beam deflection, which can more efficiently use the high photon fluxes available at the new high-brightness synchrotron sources. A further advantage as discussed in the work of Yamada *et al.* (2021 ▸), is the accuracy of the beam placement. The authors were able to estimate an upper bound to the precision of spot placement of order 1 nm based on the details of this current work, but as they point out, the potential exists to do better in subsequent experiments with some modifications.

It is important to consider a few of the new types of experiments that this work opens up. The most obvious are experiments where it is difficult to move the sample with high spatial resolution. Large-volume high-pressure presses, and heavy ultra-high-vacuum chambers fall into this category. One of the experimental difficulties for conventional SXM is that very small precise motions for big and heavy equipment are typically slow, and slow data acquisition is an inefficient use of the high-brightness synchrotron sources.

Another class of experiments that are difficult for conventional SXMs are *in-situ* experiments where the sample is either very hot, or very cold. In both cases, one needs weak thermal links in order to keep the sample at temperatures of interest, and weak thermal links are typically not mechanically rigid. For example, some cryostats that operate at milli-kelvin temperatures, have internal cold stages which are suspended by Kevlar threads under tension (Woodcraft *et al.*, 2009 ▸). The positions of samples in these cryostats will stabilize with time, but it is much more time efficient to scan the X-ray beam in this case.

This seminal work naturally leads one to consider future directions. One question is whether other types of focusing optics, such as zone plates or MLLs would have a similar or wider angular field of view to enable prism-based scanning. Another direction would be to expand the field of view beyond the size enabled by just the prism scanning. One could have a hybrid motion of both beam and sample, where one has fast prism-based motion of the beam within a field, followed by bigger sample motion steps of order the field size, which can be slower. One then ‘stitches’ the different fields together into a bigger field of view. This is analogous with electron beam lithography, where there is a high-resolution scan field within which the electron beam is scanned quickly electrostatically, and precision stage motions which are slower, allowing one to ‘stitch’ the fields together to obtain larger lithographic patterns than a single field. Finally, one needs to explore other types of prism and scanner mechanics to enhance both the speed and precision of the scanned X-ray beam.
